# Catheter ablation for atrial fibrillation in heart failure with reduced ejection fraction: a systematic review and meta-analysis of randomized controlled trials

**DOI:** 10.1186/s12872-019-0998-2

**Published:** 2019-01-15

**Authors:** Ahmed AlTurki, Riccardo Proietti, Ahmed Dawas, Hasan Alturki, Thao Huynh, Vidal Essebag

**Affiliations:** 1 Division of Cardiology, McGill University Health Center, 1650 Cedar Ave, Room E5-200, Montreal, QC H3G 1A4 Canada; 2 Department of Cardiac, Thoracic, and Vascular Sciences, Padua, Italy; 3 School of Medicine and Medical Science, University College, Dublin, Ireland; 4 Hôpital Sacré-Coeur de Montréal, Montreal, Quebec, Canada

**Keywords:** Catheter ablation, Atrial fibrillation, Heart failure

## Abstract

**Background:**

Previous randomized controlled trials (RCT)s showed similar outcomes in patients with atrial fibrillation (AF) and heart failure with reduced ejection fraction (HFrEF) treated with anti-arrhythmic drugs (AAD) compared to rate control therapy. We sought to evaluate whether catheter ablation is superior to medical therapy in patients with AF and HFrEF.

**Methods:**

We searched electronic databases for all RCTs that compared catheter ablation and medical therapy (with or without use of AAD). We used random-effects models to summarize the studies. The primary end-point was all-cause mortality. Secondary outcomes included heart failure-related hospitalizations and change in left ventricular ejection fraction (LVEF).

**Results:**

We retrieved and summarized 7 randomized controlled trials, enrolling 856 patients (429 in the catheter ablation arm and 427 in the medical therapy arm). Compared with medical therapy (including use of AAD), AF catheter ablation was associated with a significant reduction in mortality (risk ratio 0.50; 95% confidence interval [CI]: 0.34 to 0.74; *P* = 0.0005) and heart failure-related hospitalizations (risk ratio 0.56; 95% CI: 0.44 to 0.71; *P* < 0.0001). Furthermore, catheter ablation led to significant improvements in LVEF (weighted mean difference, 7.48; 95% CI: 3.71 to 11.26; *P* < 0.0001).

**Conclusions:**

Compared to medical therapy, including use of AAD, catheter ablation for AF was associated with a significant reduction in mortality and heart failure-related hospitalizations as well as an improvement in LVEF in patients with HFrEF. Larger trials are needed to confirm whether rhythm control with ablation is superior to rate control in patients with AF and heart failure.

## Introduction

Atrial fibrillation (AF) and heart failure with reduced ejection fraction (HFrEF) are two of the most commonly encountered cardiac diseases and are inextricably linked [1]. They often occur concurrently [2], with each condition perpetuating the other and are both associated with significant morbidity and mortality [3]. AF may perpetuate HFrEF through decreased cardiac output, worsening the neurohormonal response, functional mitral annular enlargement with resultant mitral regurgitation as well as by tachycardia-induced cardiomyopathy [4–8].

Rhythm control with anti-arrhythmic drugs (AADs) did not improve outcomes compared to rate control in the AFFIRM trial [9]. Two large trials were performed comparing AAD to rate control in patients with AF and HFrEF [10, 11]. In the AF-CHF trial, compared to rate control rhythm control with amiodarone did not reduce cardiovascular mortality or hospitalization [11]. In the DIAMOND trial, dofetilide resulted in greater cardioversion to and maintenance of sinus rhythm compared to rate control medical therapy. Although dofetilide did not reduce mortality, patients in whom sinus rhythm was restored and maintained had improved survival compared to the other patients who remained in AF [10].

The degree of rhythm control achieved with AADs is suboptimal and AADs may have several side effects [12, 13]. Catheter ablation is superior to AADs in providing rhythm control in patients with AF [14–17]. In addition, catheter ablation has been shown to improve functional status and quality of life as well as reduce hospitalizations [14, 18] and health resource utilization [19]. Furthermore, catheter ablation is a relatively safe procedure with a low incidence of major adverse events [20]. The potential benefit of catheter ablation in patients with AF and HFrEF has not been fully elucidated. Several observational studies of catheter ablation in patients with HFrEF reported that maintenance of sinus rhythm by catheter ablation can improve left ventricular ejection fraction, functional status as well as reduce heart failure hospitalizations [21–24]. In a meta-analysis of observational studies, catheter ablation resulted in improved LVEF compared to rate control [25].

We aimed to assess the efficacy and safety of catheter ablation compared to medical therapy in patients with AF and HFrEF by performing a systematic review and meta-analysis of randomized controlled trials.

## Methods

### Search strategy

This systematic review was performed according to the guidelines described in the PRISMA (Preferred Reporting Items for Systematic Reviews and Meta-Analyses) statement. We searched PubMed, Embase, and Cochrane Central Register of Clinical Trials using the terms: atrial fibrillation, persistent atrial fibrillation, ablation, catheter ablation, pulmonary vein isolation, heart failure, heart failure with reduced ejection fraction, congestive heart failure, left ventricular dysfunction, impaired left ventricular systolic function, reduced left ventricular systolic function, low ejection fraction, functional capacity, and quality of life. Our search was limited to human studies in peer-reviewed journals from inception to February 26th, 2018. No language restriction was applied. Hand searching with cross-references of retrieved publications, review articles and guidelines was also performed to ensure the inclusion of all relevant studies.

### Study selection

The studies had to fulfil the following criteria to be included in the analysis: (i) the study was a randomized controlled trial; (ii) the intervention arm was composed of patients undergoing radio-frequency ablation or cryoablation for AF; (iii) included a control group of medical therapy. The medical therapy control group could be either rate control with medications or atrioventricular nodal ablation with pacing, or rhythm and rate control with amiodarone (iv) follow-up duration of at least six months; and (v) reported at least one outcome of interest. The use of amiodarone was included given guideline recommendations for its possible use for rate control in addition to its role as the main anti-arrhythmic drug for rhythm control in patients with HFrEF [26, 27].

### Data extraction

Two investigators (A.A.T. and A.D.) independently performed the initial screening of titles and abstracts to identify potentially relevant articles. Review articles, case reports, meeting abstracts and duplicates were excluded. The full-text of selected articles was independently assessed by two inBCD90998vestigators (A.A.T. and A.D.) to determine relevance for inclusion. Conflicts were resolved by consensus and if necessary by additional discussion with a third author (R.P.). Extracted study and patient characteristics included data regarding study characteristics, baseline patient characteristics, procedural characteristics as well as the number of events for categorical outcomes in both arms and means and standard deviations for continuous outcomes. If this information was not reported in the published article, we contacted the corresponding author of the study for further details.

### Quality assessment

We evaluated quality of included RCTs by using the Risk of Bias Tool developed by the Cochrane Collaboration. For each RCT, 2 reviewers (A.A.T. and A.D.) independently assigned a score of high, low, or unclear to each of the following domains: sequence generation; allocation concealment; blinding of participants, personnel, and outcome assessors; incomplete outcome data; selective outcome reporting; and other potential sources of bias. Disagreements were resolved by consensus. We included all eligible RCTs regardless of their assessed quality.

### Primary and secondary outcomes

The primary end-point was all-cause mortality. Secondary outcomes included heart failure hospitalizations, change in LVEF, change in six-minute walk test distance and change in Minnesota living with heart failure (MLWHF score). Outcomes were collected at the end of study follow-up.

### Statistical analysis

Descriptive statistics are presented as means and standard deviations (SD) for continuous variables or number of cases (n) and as percentages (%) for dichotomous and categorical variables. Publication bias was assessed visually using a funnel plot and quantified using Egger’s test for small study effects. Heterogeneity among studies was assessed using the inconsistency index (I^2^) statistic, which ranges from 0 to 100%. I^2^ is defined as the percentage of the observed inter-trial variability that is due to heterogeneity (true difference between trials) rather than chance for each outcome; I^2^ > 50% denotes significant heterogeneity. The results are presented as risk ratios with 95% confidence intervals (CIs) for calculated categorical outcomes using the total number of events reported in each included trial. For continuous outcomes weighted mean differences with 95% CIs were used using the means and standard deviations reported in each included trial. Data used was based on an intention to treat analysis. Random-effect models were used for all reported outcomes. All statistical analyses were performed with the use of STATA software version 14.3 (College Station, TX: StataCorp LP) and Statsdirect version 3 (England: StatsDirect Ltd. 2013). Two-tailed probability values of < 0.05 were considered significant.

### Sensitivity analysis

Influence analyses using random-effects models were performed to assess the effect of each trial on metanalytic results. In addition, we performed a sensitivity analysis excluding trials that allowed AAD therapy in the comparison group. For LVEF assessment, we performed sensitivity analyses restricting follow up to 6–12 months as well as excluding the study that used a LVEF of less than 50% as an entry cut-off.

## Results

### Study selection

**Fig. 1:**
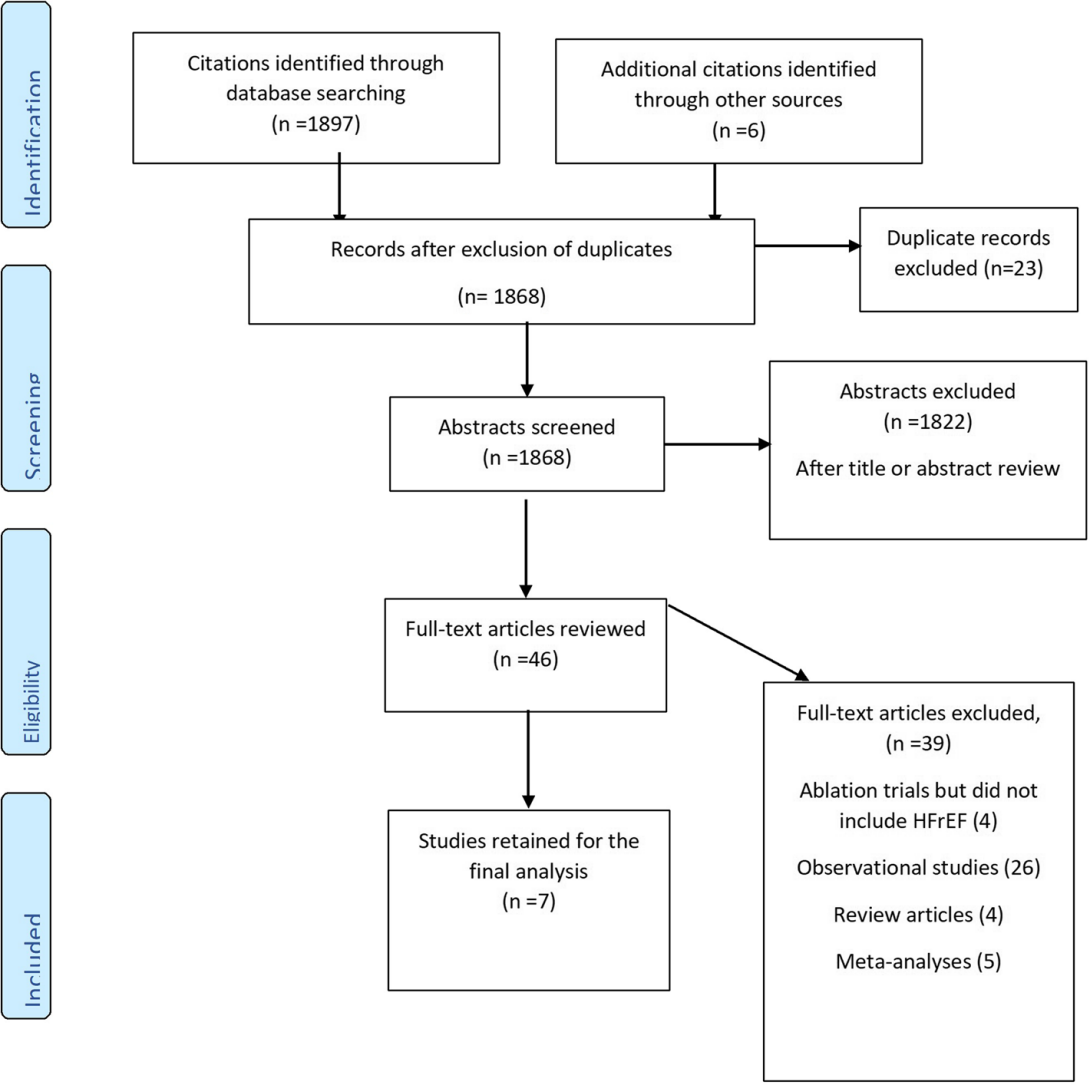
Preferred reporting items for systematic reviews and meta-analyses flow diagram

The search strategy identified 1868 abstracts, out of which 1822 were removed after title and abstract review (Fig. 1). Forty-six full-text manuscripts were assessed for eligibility. Seven studies (Table 1) fulfilled the inclusion criteria and were included in the present meta-analysis [28–34]. Thirty-nine studies were excluded from the final analysis because they did not meet the inclusion criteria: 30 were observational studies, four were review articles and five were meta-analyses.

**Table 1:** Study characteristics of the included randomized controlled trials comparing atrial fibrillation catheter ablation to medical therapy in patients with heart failure and reduced ejection fraction

Study (year)	N (ablation/ medical therapy)	Setting (centers)	Type of AF	Mean follow-up (months)	Heart rhythm assessment modality	Frequency of rhythm monitoring (months)	Ablation technique	Ablation strategy	Medical therapy	Primary Outcome	Quality^a^
Khan (2008)[31]	41/40	Multi	Persistent 50% Paroxysmal 50%	6	Loop recorder	2, 3, 6	RF	PVI ± Linear lesions & CFAE	AVN ablation+BiV pacing	Change in LVEF, 6- min walk distance and MLWHF score	Fair
Macdonald (2010)	22/19	Single	Persistent	6	24-h Holter monitor	3, 6	RF	PVI ± Linear lesions & CFAE ± CVTI (+ 3 months amiodarone)	Rate control with BB ± Dig	Change in LVEF	Good
Jones (2013)[30]	26/26	Multi	Persistent	12	48-h Holter monitor	2, 3, 6, 12	RF	PVI ± Linear lesions ± CFAE ± CVTI	Rate control with BB ± Dig	Change in peak oxygen consumption	Fair
Hunter (2014)[29]	26/24	Single	Persistent	6	48-h Holter monitor	1, 3, 6	RF	PVI with CFAE ± Linear lesions ± CVTI	Rate control	Change in LVEF	Good
Di Biase (2016)[28]	102/101	Multi	Persistent	24	ICD/CRT-D	3, 6, 12, 24 (device interrogation)	RF	PVI + LAPWI+ SVCI+ CFAE	Amiodarone	AF recurrence	Good
Prabhu et al. 2017[34]	33/33	Multi	Persistent	6	Loop recorder	3, 6	RF CF	PVI + LAPWI	Rate control	Change in LVEF	Good
Marrouche (2018)[33]	179/184	Multi	Persistent 70% Paroxysmal 30%	38	ICD/CRT-D	3, 6, 12, 24, 36, 48, 60 (device interrogation)	Operator discretion	PVI + Operator discretion	Rate or Rhythm control	Mortality and heart failure hospitalization	Fair

*N* number, *AF* atrial fibrillation, *RF* radiofrequency, *PVI* pulmonary vein isolation, *CFAE* complex fractionated atrial electrogram, *AVN* atrioventricular node, *BiV* biventricular, *LVEF* left ventricular ejection fraction, *MLWHF* Minnesota living with heart failure, *CVTI* cavotricuspid isthus, *BB* beta blocker, *ICD* implantable cardioverter-defibrillator, *CRT-D* cardiac resynchronization therapy- defibrillation, *LAPWI* left atrial posterior wall isolation, *SVCI* superior vena cava isolation. ^a^Using the Cochrane risk of bias tool

### Quality assessment

**Fig. 2:**
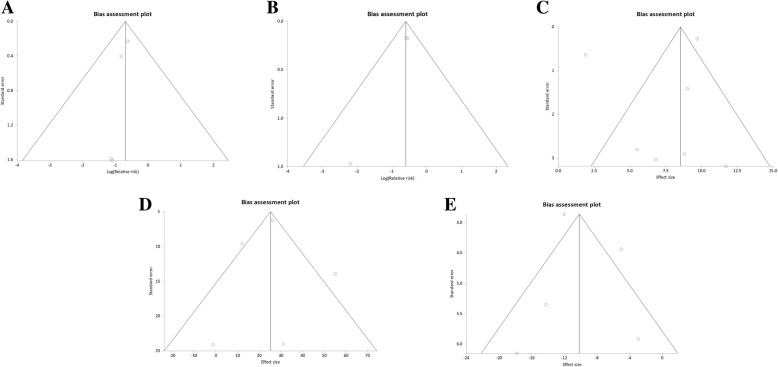
Funnel plot for (**a**) mortality, (**b**) heart failure-related hospitalization, (**c**) change in left ventricular ejection fraction, (**d**) change in six-minute walk test, (**e**) change in Minnesota living with heart failure questionnaire score

Overall there was a low risk of bias. The risks of bias of the included studies are shown in Table 2. No publication bias was suggested by the funnel plots or Egger’s test (Fig. 2 and Table 3). All were multicenter trials done according to the intention-to-treat principle.

**Table 2:** Risk of publication bias

Study (year)	Random sequence generation	Allocation concealment	Blinding of participants and personnel	Blinding of outcome assessment	Incomplete outcome data	Selective reporting	Other sources of bias
Khan (2008)[31]	+	?	–	+	+	+	+
Macdonald (2010)[32]	+	+	–	+	+	+	+
Jones (2013)[30]	+	?	–	+	+	+	+
Hunter (2014)[29]	+	+	–	+	+	+	+
Di Biase (2016)[28]	+	?	–	+	+	+	+
Prabhu et al. 2017[34]	+	?	–	+	+	+	+
Marrouche (2018)[33]	+	?	–	–	+	+	+

“+” = low bias risk; “-”= substantial bias risk; “?” = unclear bias risk

**Table 3:** Egger’s test for bias

	Egger’s bias	95% confidence interval	*P*-value
Mortality	0.41	−1.14 to 0.32	0.14
Heart failure related hospitalization	−1.46	−4.49 to 1.55	0.15
Left ventricular ejection fraction	1.58	−8.01to 4.83	0.55
Six-minute walk test	0.07	−4.81 to 4.94	0.97
Minnesota living with heart failure score	0.62	−11.01 to 9.78	0.86

### Patient characteristics

The efficacy and safety of catheter ablation of AF in HFrEF was analyzed in seven randomized controlled studies that enrolled 856 patients including 429 in the catheter ablation arm and 427 in the rate control arm [28–34]. The mean age of patients included in the trials ranged from 57.4 ± 11.0 to 64 ± 8 years. The proportion of men in the studies was 84.4%. The mean proportion of patients with ischemic cardiomyopathy was 47.6%. Mean LVEF was 28% and mean NYHA class was 2.5. Baseline patient characteristics are summarized in Table 4.

**Table 4:** Baseline patient characteristics

Study (year)	Catheter Ablation										Medical Therapy									
	Age (mean, years)	Sex (F) (%)	HTN (%)	DM (%)	CAD (%)	NCMP (%)	LAS (mean, cm)	BB (%)	EF (mean)	NYHA class (mean)	Age (mean, years)	Sex (F) (%)	HTN (%)	DM (%)	CAD (%)	NCMP (%)	LAS (mean, cm)	BB (%)	EF (mean)	NYHA class (mean)
Khan (2008)[31]	60	5	NA	NA	73	27	4.9	NA	27	NA	61	12	NA	NA	68	32	4.7	NA	29	NA
Macdonald (2010) [32]	62	23	64	32	50	50	NA	82	NA	NA	64	21	58	21	47	53	NA	95	NA	NA
Jones (2013)	64	19	NA	NA	42	62	5.0	92	22	2.46	62	8	NA	NA	50	73	4.6	92	25	2.5
Hunter (2014)[29]	55	4	31	NA	23	77	5.2	NA	32	2.6	60	4	33	NA	29	71	5.0	NA	34	2.5
Di Biase (2016)[28]	62	25	45	22	62	38	4.7	76	29	NA	60	27	48	24	65	35	4.8	81	30	NA
Prabhu et al. 2017[34]	59	6	39	12	NA	NA	4.8	97	35	2.55	62	12	36	15	NA	NA	4.7	97	35	2.45
Marrouche (2018)[33]	64	13	72	24	28	59	4.8	93	33	2.41	64	16	74	36	36	49	5.0	94	32	2.43

*F* female, *HTN* hypertension, *DM* diabetes mellitus, *CAD* coronary artery disease, *NCMP* non-ischemic cardiomyopathy, *LAS* left atrial size, *BB* beta blocker, *EF* ejection fraction, *NYHA* New York Heart Association, *NA* not available

### Catheter ablation protocols

All studies used radiofrequency ablation exclusively except for one trial which left the ablation system to the discretion of the operator. One study incorporated contact force technology. Pulmonary vein isolation was the standard ablation strategy used. Six studies used additional linear lesions [28–32, 34], with two studies aiming for left atrial posterior wall isolation [28, 34], while in the remaining study the entire strategy was left to the discretion of operators, who were required to have performed at least 50 ablations previously [33].

### Arrhythmia free survival

We assessed arrhythmia-free survival at the end of follow-up as the measure of success of catheter ablation in maintaining sinus rhythm. Six of the seven included trials reported arrhythmia-free survival. With the exception of the study by McDonald et al. [32], which reported an arrhythmia free survival of 50%, all other studies reported an arrhythmia free survival of over 70% at the end of follow-up [28–31, 33, 34]. The highest reported survival was 92% in the study reported by Hunter et al. [29]. At the longest reported follow-up, 37 months in CASTLE-AF, the arrhythmia free-survival rate was 75% in patients who underwent catheter ablation.

### Clinical outcomes

**Fig. 3:**
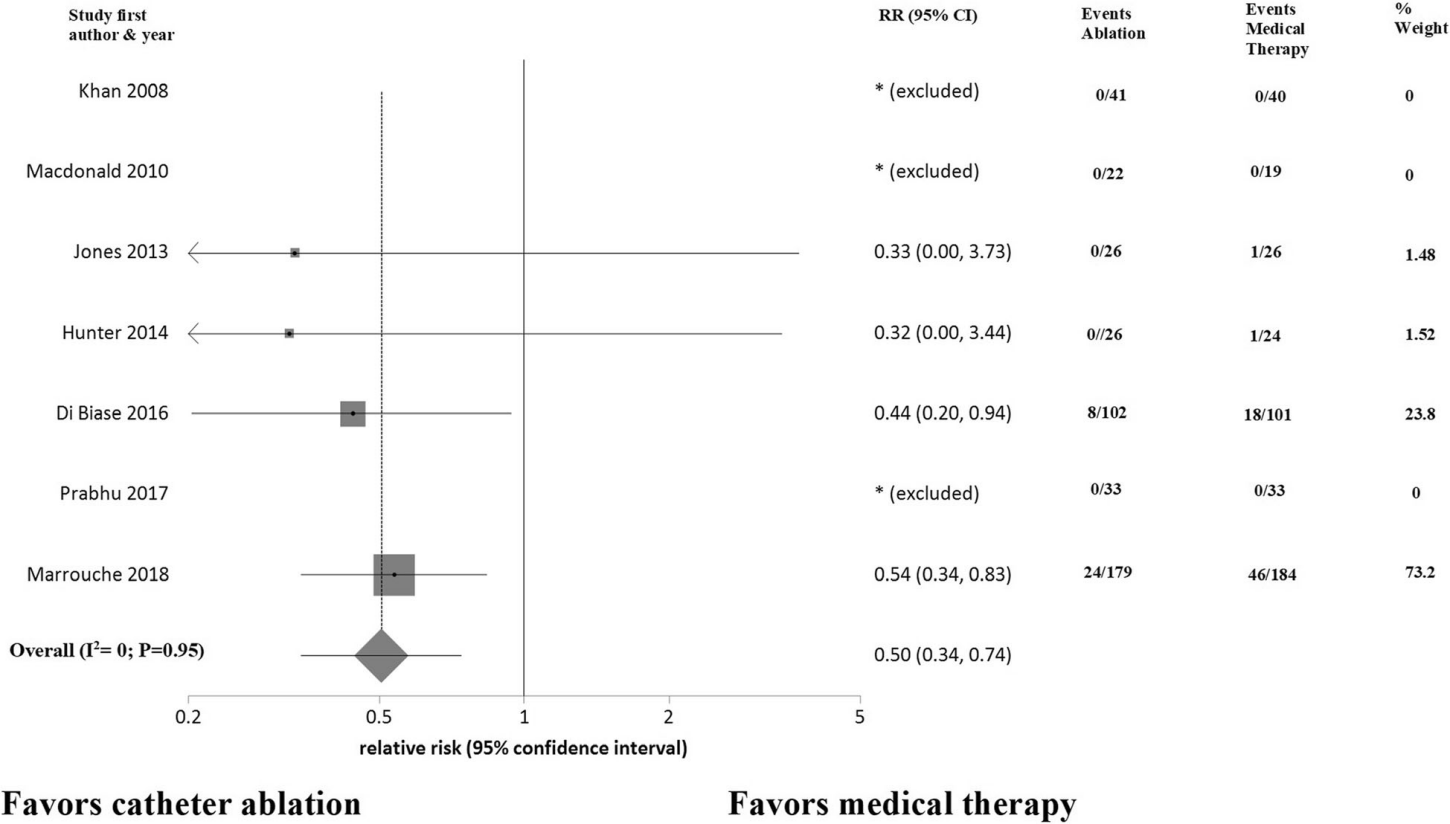
Forest plot showing random effects summary of all-cause mortality

**Fig. 4:**
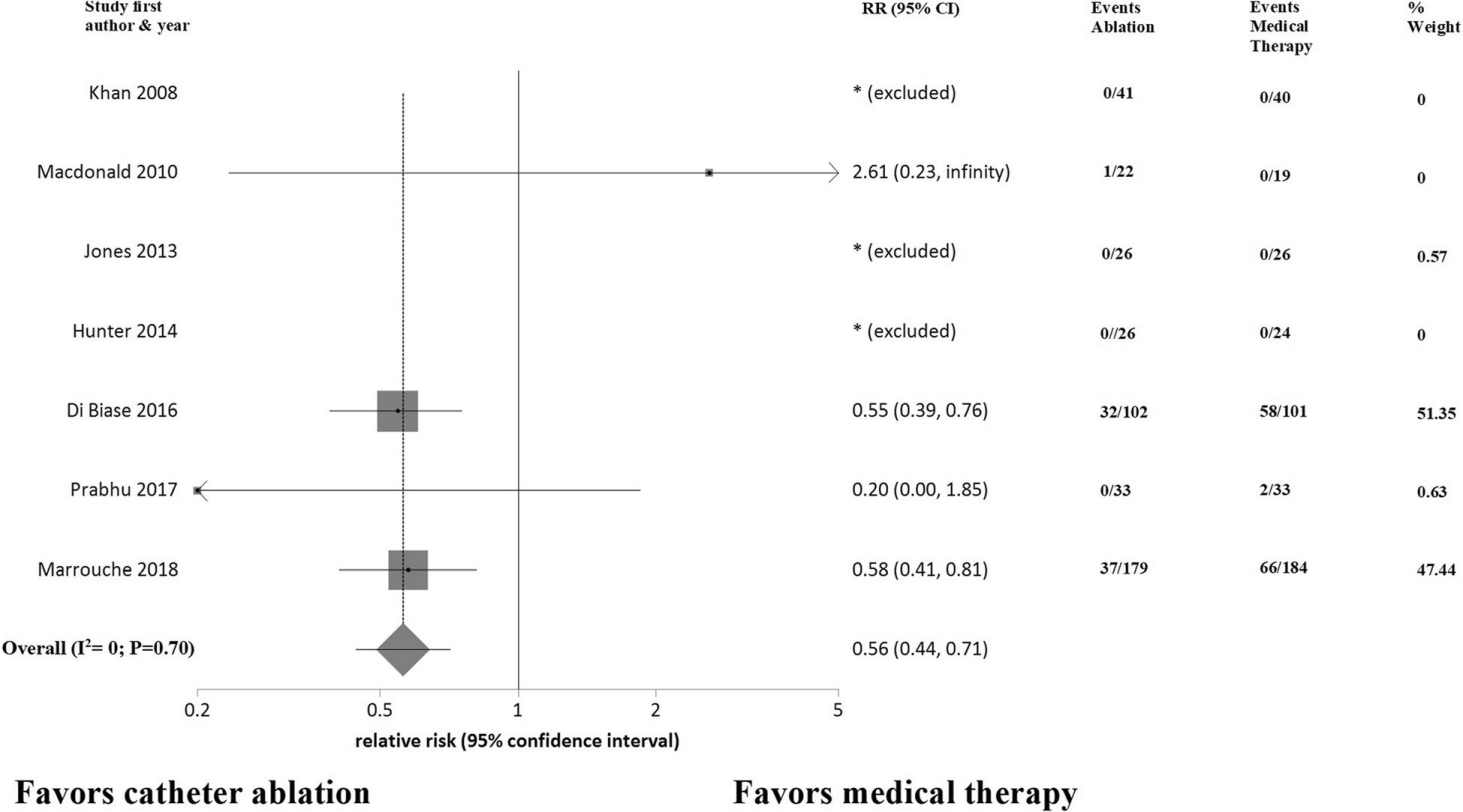
Forest plot showing random effects summary of heart failure hospitalization

All trials reported data on clinical outcomes, mortality and heart failure hospitalization, at the end of study follow-up. AF catheter ablation was associated with a significant reduction in mortality (risk ratio [RR] 0.50; 95% CI: 0.34 to 0.74; *P* = 0.0005) (Fig. 3) and heart failure hospitalizations (RR 0.56; 95% CI: 0.44 to 0.71; *P* < 0.0001) (Fig. 4) compared to a medical therapy strategy. No heterogeneity was detected (I^2^ = 0%) and there was no evidence of publication bias.

### Left ventricular ejection fraction

**Fig. 5:**
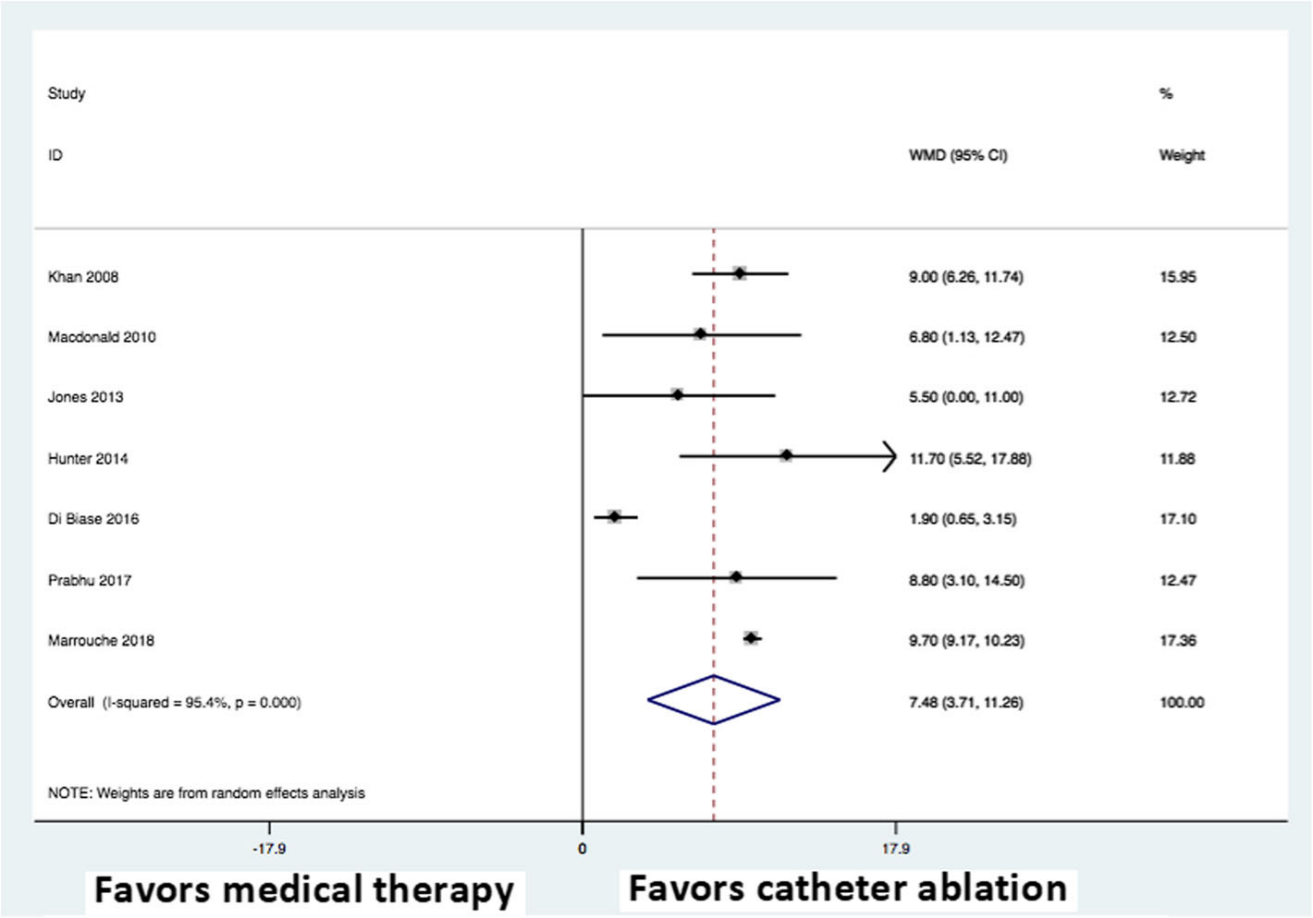
Forest plot demonstrating random effects summary of change in LVEF

Al trials reported data on change in LVEF. Compared with medical therapy including AAD, AF catheter ablation was associated with a significant increase in LVEF (weighted mean difference, 7.48; 95% CI: 3.71 to 11.26; *P* < 0.0001) (Fig. 5). These results were consistent across all seven trials. There was significant heterogeneity (I^2^ = 97%) but no evidence of publication bias. When restricted to 6–12 month follow up, catheter ablation was also associated with an increase in LVEF (weighted mean difference, 7.00; 95% CI: 5.02 to 8.99; *P* < 0.0001); there was no heterogeneity (I^2^ = 0%) when follow-up was restricted to 6–12 months. When only pure rate control strategies were considered (exclusion of comparison with any amiodarone use in Di Biase et al. [28] and Marrouche et al. [33]), five trials reported data and catheter ablation was associated with an increase in LVEF (weighted mean difference, 8.53; 95% CI: 6.54 to 10.51; *P* < 0.0001; I^2^ = 0%).

### Functional capacity and quality of life

**Fig. 6:**
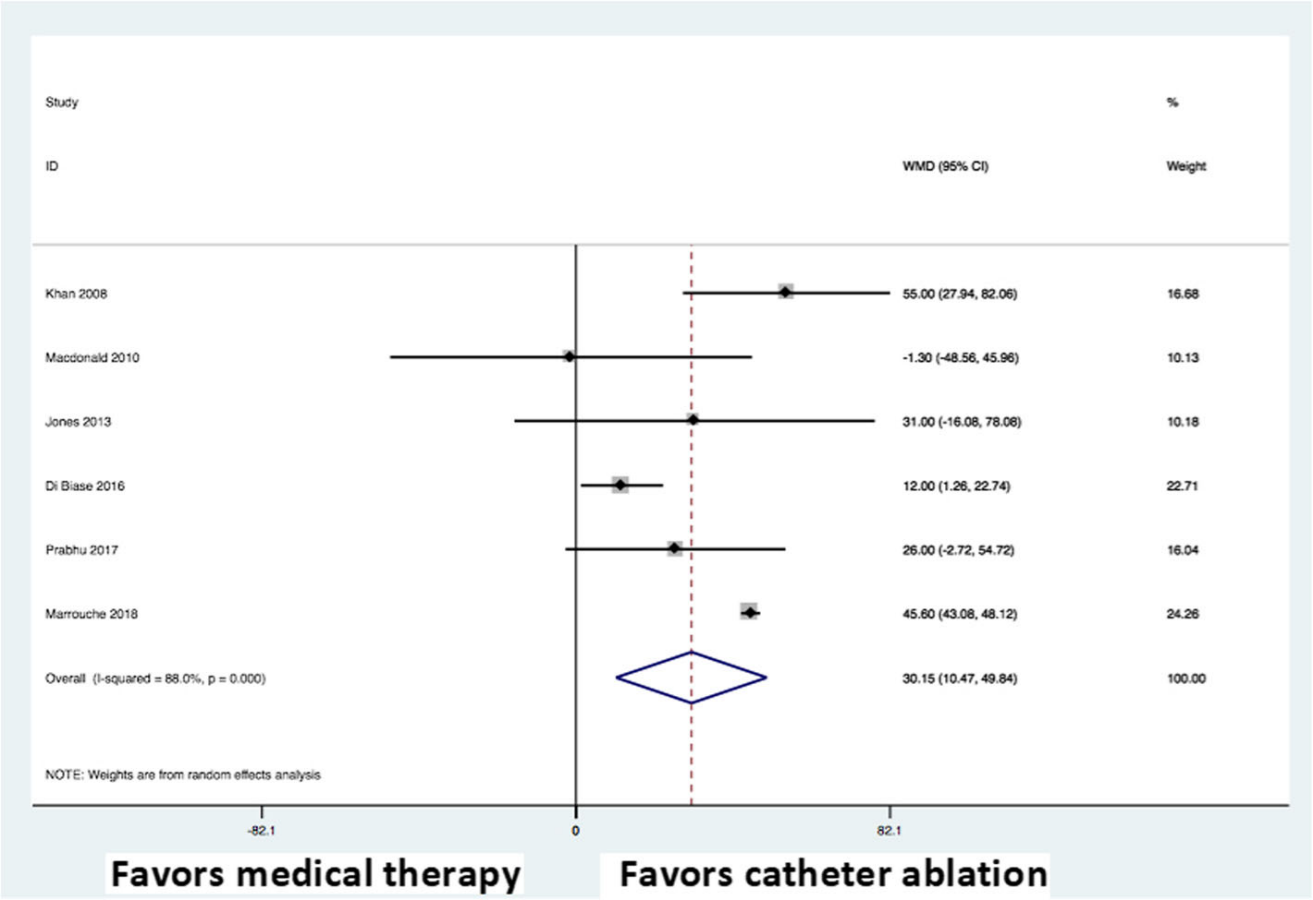
Forest plot showing random effects summary of change in six-minute walk test distance

**Fig. 7:**
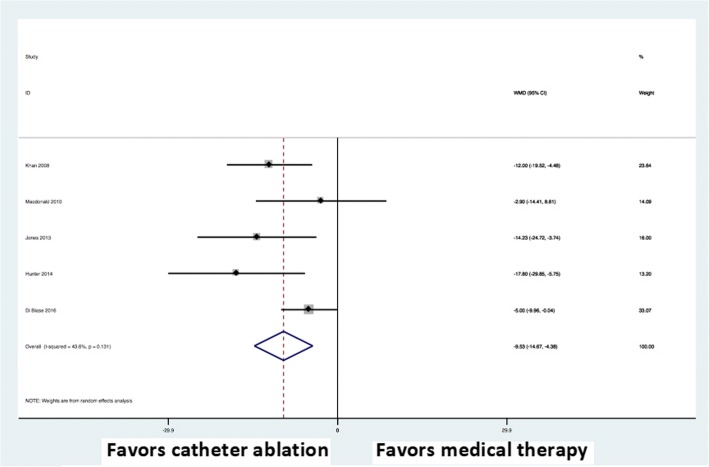
Forest plot showing random effects summary of change in Minnesota Living With Heart Failure Questionnaire score

All trials reported on functional capacity using the 6MWT and five trials reported on quality of life using the MLWHF questionnaire. There was a significant improvement in 6MWT performance (weighted mean difference, 30.15; 95% CI: 10.47 to 49.84; P < 0.0001) (Fig. 6) and MLWHF questionnaire scores (weighted mean difference, − 9.53; 95% CI: –14.67 to − 4.38; P < 0.0001) (Fig. 7) in the AF catheter ablation group versus the medical therapy group.

### Complications

Peri-procedural complications were reported by all seven trials. The overall incidence of was 7.3% (95% CI 3.4–11.3%), with bleeding complications occurring in 2.4% of the patients (95% CI 0.9–3.9%). Bleeding complications occurred in eight patients with pericardial effusion; five required immediate intervention and three patients with groin hematoma. Ablation related complications are summarized in Table 5.

**Table 5:** Peri-procedural complications

Study (year)	Pericardial effusion	Cerebrovascular events (stroke or TIA)	Groin hematoma	HF exacerbation	Pneumonia
Khan (2008)[31]	1	0	3	1	0
Macdonald (2010)	2 (tamponade required pericardiocentesis)	1	0	3	1
Jones (2013)	1 (tamponade requiring sternotomy)	0	1	1	1
Hunter (2014)[29]	1 (tamponade)	1	0	0	0
Di Biase (2016)[28]	1 (requiring FFP and protamine sulphate)	0	2	0	0
Prabhu et al. 2017[34]	0	0	1 (requiring transfusion)	0	1
Marrouche (2018)[33]	3 (1 required pericardiocentesis)	8	3	1	3

*TIA* transient ischemic attack, *FFP* fresh frozen plasma

### Sensitivity analysis

The sensitivity analysis was consistent with the base-case analysis. An influence analysis with sequential exclusion of each study did not change the study outcomes of mortality, heart failure-related hospitalization, LVEF, 6MWT and MLWHF score. When trials that allowed AAD use in the medical therapy group were excluded (Di Biase et al. [28] and Marrouche et al. [33], there was no significant difference in mortality or heart failure-related hospitalization (although limited sample size with very low number of events to compare). In contrast, the improvement in LVEF, 6MWT and MLWHF score persisted.

## Discussion

The primary finding of this meta-analysis, of patients with AF and ambulatory HFrEF with a mean NYHA of 2.5, is a significant reduction in all-cause mortality and heart failure hospitalizations in patients undergoing catheter ablation compared to those who receive medical therapy including AAD. In addition, there was a significant improvement in LVEF in patients who undergo catheter ablation. This benefit was consistent and similar in magnitude across all included trials including those with longer term follow up [28–34]. There was no heterogeneity in clinical outcomes but significant heterogeneity in LVEF and 6MWT assessment. This likely reflects the heterogeneity in testing as well as the varying degrees of follow-up. However, in all the included studies, the results for clinical outcome and change in LVEF functional assessment was consistently in favor of catheter ablation. The results of this study are in keeping with the current guidelines that state that catheter ablation of AF in patients with HF is an effective and acceptable therapeutic option and recommend its use for similar indications as for patients without HFrEF [35].

The deleterious effects of AF are well established, especially the increased risk of stroke. Recent evidence has shown that the complications of AF extend beyond stroke and include an increased risk of mortality, myocardial infarction as well as increased heart failure incidence and hospitalizations. In a large population-based administrative database, Ionescu-Ittu et al. showed that patients with a rhythm control strategy for AF had lower mortality over long-term follow-up [36]. In a large prospective cohort study of 15,400 patients with AF, 11% died at one-year follow-up, with heart failure being the commonest cause of death [37]. In a large meta-analysis of 104 studies involving 9,686,513 patients of whom 587,867 had AF, the presence of AF was associated with a two-fold increase in mortality and a five-fold increase in heart failure [38]. Therefore, given the increased risk of cardiovascular events in the HFrEF population and AF [39], the current meta-analysis suggest that restoring sinus rhythm by catheter ablation reduces mortality and heart failure-related hospitalizations.

The beneficial effect of catheter ablation on LVEF has been consistent across meta-analyses of observational studies and small randomized studies [25, 40]. We demonstrated a mean difference in LVEF improvement of 7.5%. This improvement was consistent across all RCTs and only marginally lower than 11.0% improvement seen in observational studies [25]. As mentioned, the high degree of heterogeneity is likely due to variants in LVEF measurement methods which may be observer dependent as well as due to the differences in duration of follow-up. This was corroborated by the analysis of the 5 trials with 12 months or less of follow-up which showed a similar improvement in LVEF but no heterogeneity. An influence analysis with exclusion of each trial did not change the results of our analysis. Furthermore, the similar results with exclusion of studies that used amiodarone or AV node ablation with ventricular pacing supported the validity of our results. While one study suggested an improvement in LVEF with pharmacological rhythm control [41], this was not corroborated in other studies [42, 43] nor in a meta-analysis [44]. Our finding of consistent improvement in LVEF with catheter ablation in all the RCTs evaluated is likely explained by the superiority of catheter ablation in maintenance of sinus rhythm in comparison to anti-arrhythmic drugs for LVEF [45]. Furthermore, the correlation between improvement in LVEF and clinical outcomes with catheter ablation, as well as the lack of improvement of LVEF and clinical outcomes with AAD, reinforces the value of LVEF as a surrogate marker for clinical outcomes [46, 47].

Interestingly, the proportion of patients who remained in sinus rhythm was relatively high especially considering that most patients had prior persistent AF. The proportions of patients who remained in sinus rhythm ranged from 80 to 90% at one year follow up to 70 and 63% at two and five years follow up respectively. This likely reflects the considerable improvement in technique and the ability of catheter ablation to maintain sinus rhythm even in cases with HFrEF and persistent AF. Furthermore, inasmuch as presence of heart failure favors maintenance of AF [48], the improvement in heart failure with restoration of sinus rhythm may also contribute to success of ablation by modifying the underlying electro-anatomical substrate [49] (i.e. possibly to a greater extent than achievable by ablation of long-standing persistent AF without heart failure). Success rates of catheter ablation of long-standing persistent AF without HFrEF, in a single center study, were 20 and 45% after single and multiple ablations respectively at five-year follow up [50]. Maintenance of sinus rhythm with AAD is especially difficult in HFrEF. In the AF-CHF trial, 21% abandoned rhythm control, 27% were in AF at the end of the 4-year follow up and 58% had experienced at least one episode of AF [11]. Loss of the atrial systole, a decreased diastolic filling interval and increased neurohormonal activation may be of particular hemodynamic consequence in this population [48]. In the DIAMOND AF study, patients who were able to maintain sinus rhythm had improved outcomes [10].

We found that catheter ablation is a safe procedure in patients with AF and HFrEF. The number of adverse events were relatively small and are comparable to those seen in patients with paroxysmal AF. In addition, the very low degree of side effects renders catheter ablation an attractive option for maintenance of sinus rhythm especially considering the low efficacy rate and higher degree of side effects of AADs.

Similar to a previous meta-analysis of randomized trials comparing AF catheter ablation to anti-arrhythmic drug therapy in patients without HFrEF [51], the current analysis demonstrated an improvement in functional status and quality of life in patients with HFrEF. Given the non-blinded nature of the intervention, the possibility that the improvements in functional status be due to a placebo effect cannot be excluded. However, the improvement in functional status mirrors that seen in LVEF and clinical outcomes. This association is well established in patients with HFrEF [52]. Furthermore, the six-minute walk distance is a recognized objective measure of functional status [53].

Of note, a major limitation of this analysis and the current literature is the absence of clinical trials with long-term clinical outcome assessment comparing catheter ablation with a pure rate control strategy i.e. excluding amiodarone use completely. As seen in the AF-CHF trial [11], use of amiodarone in the rhythm control group may have offset the benefit achieved from a relatively good degree of maintenance of sinus rhythm. Hence the RAFT-AF randomized trial (NCT01420393), which will assess the long-term outcome of catheter ablation in comparison to rate control therapy, excluding AAD use such as amiodarone, will shed important light on this important issue.

There were several other limitations to our meta-analyses. Firstly, the number of included studies and patients is relatively small, emphasizing the need for larger studies in this patient population. The small number of studies prevents an effective analysis of publication bias and meta-regression to better understand the factors associated with these results. Secondly, the absence of patient-level data prevented us from identifying patient characteristics that can be associated with improved outcomes. Thirdly, there was a significant variation in follow-up duration with only two studies with more than one year of follow-up. Therefore, we could not evaluate the benefit of catheter ablation beyond one-year follow-up. Fourthly, the mean NYHA class was 2.5 and the mean LVEF was 30%; therefore, it is unclear if these findings are applicable to patients with severely decreased LVEF and those with NYHA class III-IV. Fifthly, all patients included in the two largest trials reported by Di Biase et al. and Marrouche et al. included patients with an implantable-cardioverter-defibrillator [28, 33]. While this therapy is guideline based and allows continuous monitoring for arrhythmia recurrence, it remains unclear if catheter ablation is also beneficial in patients without an implantable-cardioverter-defibrillator especially given that it may no longer be indicated if the LVEF improves after catheter ablation. This clinical question should also be answered by the RAFT-AF study (NCT01420393). Data from the CABANA study (NCT00911508) specific to the heart failure population will also offer further insights. Finally, the results of our findings may only be applicable to large-volume centers of catheter ablation since most of the centers participating in the RCTs were institutions with expertise in this technique.

## Conclusions

Compared to medical therapy including AAD, AF catheter ablation was associated with significant improvements in all-cause mortality, heart failure hospitalization, LVEF as well as functional status in patients with HFrEF and AF. Larger multicenter RCTs are needed to validate whether a rhythm control strategy with AF ablation is superior to rate control strategy (without use of AAD) in patients with AF and heart failure.

## Abbreviations

AAD: Anti-arrhythmic drug
AF: Atrial fibrillation
CA: Catheter ablation
HFrEF: Heart failure with reduced ejection fraction
LVEF: Left ventricular ejection fraction
MLWHF: Minnesota living with heart failure
RCT: Randomized controlled trial
